# Supervision as a tool of professional support of specialists

**DOI:** 10.1192/j.eurpsy.2023.803

**Published:** 2023-07-19

**Authors:** D. Dovbysh, M. Bebchuk

**Affiliations:** 1 Pedagogy and medical psychology, I.M. Sechenov First Moscow State Medical University; 2 Institute of Integrative Family Therapy, Moscow, Russian Federation

## Abstract

**Introduction:**

In recent years, mental health professionals have faced a lot of difficulties and challenges in their work and often need the help of colleagues themselves.

**Objectives:**

To study the specifics of self-reflection of mental health professionals in different years (January 2020 - October 2022)

**Methods:**

Since 2002, Institute of Integrative Family Therapy has been using an approved registration card, which is filled in and handed over after completion of work by all specialists working with families. The maps contain sections describing the progress of work, hypotheses, system parameters of the family, features of the state of the specialist and clients, and so on. We conducted a content analysis of the cards: in 2020, 531 cards were considered, in 2021 - 390, in 2022 - 464 cards.

**Results:**

There are differences in the subjective assessment by specialists of their condition over the years. In the description for the section “Themes and questions in family work that elicited strong emotional reactions from the therapist(s”), professionals began to mention their reactions of fear and confusion more often than before. So, in 2020, fear was mentioned by 4 and confusion by 17 specialists, in 2021 - by 5 and 16, in 2022 - by 36 and 121, respectively. In the section related to the reasons for changing the working (systemic) hypothesis, specialists changed the hypothesis more often (in 2020 - in 53 cases, in 2021 - 40, in 2022 - 98). “The degree of satisfaction of the specialist (-s) with the results of working with the family (in points from 1 to 10, where 1 - absolutely not satisfied, 10 - satisfaction exceeded all expectations" was assessed in 2022 by specialists lower than in previous years: the average value in 2020 - 8.8; in 2021 - 8.9; in 2022 - 6.2.

**Conclusions:**

Supervision, as a form of professional growth and support from a more experienced colleague, is becoming an indispensable component of the work of a specialist helping families in 2022. Assistance in overcoming “dead end” and advising difficult cases due to the experience of the supervisor, on the one hand, allows specialists to understand the situation, offer alternative hypotheses, teach new interventions, on the other hand, it helps the supervised colleague to reflect on the case, analyze its “blind” zones, understand mistakes and summarize the unique experience gained in the psychotherapy of a “difficult” patient.

**Disclosure of Interest:**

None Declared

